# Colorectal polyps increase the glycolytic activity

**DOI:** 10.3389/fonc.2023.1171887

**Published:** 2023-06-05

**Authors:** Egle Rebane-Klemm, Leenu Reinsalu, Marju Puurand, Igor Shevchuk, Jelena Bogovskaja, Kulliki Suurmaa, Vahur Valvere, Rafael Moreno-Sanchez, Tuuli Kaambre

**Affiliations:** ^1^ Laboratory of Chemical Biology, National Institute of Chemical Physics and Biophysics, Tallinn, Estonia; ^2^ Department of Chemistry and Biotechnology, School of Science, Tallinn University of Technology, Tallinn, Estonia; ^3^ Clinic of Diagnostics, North Estonia Medical Centre, Tallinn, Estonia; ^4^ Department of Gastroenterology, West Tallinn Central Hospital, Tallinn, Estonia; ^5^ Oncology and Hematology Clinic, North Estonia Medical Centre, Tallinn, Estonia; ^6^ Laboratorio de Control Metabólico, Facultad de Estudios Superiores Iztacala, Universidad Nacional Autónoma de México, Los Reyes Iztacala, Barrio de los Árboles/Barrio de los Héroes, Tlalnepantla, Mexico

**Keywords:** metabolic phenotype, energy metabolism, colorectal cancer, colonic adenoma, OXPHOS, Warburg effect

## Abstract

In colorectal cancer (CRC) energy metabolism research, the precancerous stage of polyp has remained rather unexplored. By now, it has been shown that CRC has not fully obtained the glycolytic phenotype proposed by O. Warburg and rather depends on mitochondrial respiration. However, the pattern of metabolic adaptations during tumorigenesis is still unknown. Understanding the interplay between genetic and metabolic changes that initiate tumor development could provide biomarkers for diagnosing cancer early and targets for new cancer therapeutics. We used human CRC and polyp tissue material and performed high-resolution respirometry and qRT-PCR to detect changes on molecular and functional level with the goal of generally describing metabolic reprogramming during CRC development. Colon polyps were found to have a more glycolytic bioenergetic phenotype than tumors and normal tissues. This was supported by a greater *GLUT1*, *HK*, *LDHA*, and *MCT* expression. Despite the increased glycolytic activity, cells in polyps were still able to maintain a highly functional OXPHOS system. The mechanisms of OXPHOS regulation and the preferred substrates are currently unclear and would require further investigation. During polyp formation, intracellular energy transfer pathways become rearranged mainly by increasing the expression of mitochondrial adenylate kinase (*AK*) and creatine kinase (*CK*) isoforms. Decreased glycolysis and maintenance of OXPHOS activity, together with the downregulation of the CK system and the most common AK isoforms (*AK1* and *AK2*), seem to play a relevant role in CRC development.

## 1 Introduction

Colorectal cancer (CRC) is a multifactorial and heterogeneous disease that mostly arises from precursor lesions known as polyps. Two major classes of colorectal polyps are conventional adenomas (tubular, tubulovillous, or villous adenoma) and serrated polyps (hyperplastic polyps, sessile serrated adenoma/polyps, and traditional serrated adenomas) (1), which are believed to arise from distinct etiologic pathways. The current understanding of CRC development suggests that the progressive accumulation of oncogenic changes begins with abnormal growth of colon epithelial cells. Sequence alterations in specific genes, including *APC* and *KRAS*, contribute to the development of early precancerous lesions (2) and metabolic reprogramming towards a glycolytic phenotype. Over time, adenomas develop increasingly dysplastic features and eventually acquire malignant potential. However, most adenomas stabilize their growth progression or even regress (3). Although genetic events in colonic polyps are quite well characterized (4, 5), the reprogramming of metabolic pathways has not been widely investigated.

Metabolic reprogramming is one of the hallmarks of cancer (6). However, metabolic alterations in the precancerous stage and colorectal carcinogenesis are not well understood. Almost 100 years ago, Otto Warburg first described that cancer cells metabolize glucose directly to lactic acid even in the presence of high oxygen. This modified glucose metabolism is known as the “Warburg effect” (7). Warburg proposed that the increased rate of aerobic glycolysis was due to irreversible injury of mitochondrial oxidative phosphorylation (OXPHOS), the main pathway providing energy for eukaryotic cells, and generating more adenosine triphosphate (ATP) than glycolysis. Nowadays, it has become clear that glycolysis is upregulated in many tumors without mitochondrial dysfunction.

Several *in vivo* studies have demonstrated up-regulation of the components of the OXPHOS system in certain types of cancer cells (8), which is accompanied by increased mitochondrial respiration and OXPHOS flux. The OXPHOS machinery in most cancer cells seems to be fully functional. Moreover, cells can switch between OXPHOS and aerobic glycolysis or even perform them simultaneously, depending on the availability of substrates (including oxygen) (9). This metabolic plasticity is defined as the ability of cancer cells to reprogram their metabolic pathways to fulfill energetic and anabolic needs in a changing extracellular microenvironment during the various steps of disease progression.

Another important aspect of energy metabolism and metabolic plasticity is the interplay between energy transfer pathways in cancer cells. The isoenzymes of hexokinase (HK), adenylate kinase (AK), and creatine kinase (CK) support specific cellular processes ranging from muscle contraction and cell motility to mitochondrial/nuclear energetics (10). Indeed, it has been proposed that some AK and HK isoenzymes may be targets for antitumor therapy (11, 12). A complete spectrum of HK, AK, and CK isoforms in clinically well-defined patient groups may inform us about the changes in the maintenance of energy homeostasis of tumor cells. Several high-resolution respirometry studies performed on different permeabilized tissues and cells show that there is specificity on how adenosine diphosphate (ADP) may regulate OXPHOS at the level of the mitochondrial outer membrane (MOM). The basis of this last premise is the structurally different intracellular arrangement of functional units; such complexity of the intracellular environment determines the need for the use of energy transfer pathways. Moreover, the differences between cancer and non-cancer cells in the composition of cytoskeleton proteins and their interaction with mitochondria are related to the prevalent type of metabolism, facilitating metabolic plasticity (13, 14).

The present study was aimed to identify and characterize metabolic reprogramming in colon polyps by assessing mitochondrial respiratory rates and gene expression of selected metabolic markers. The precise contribution of different metabolic pathways to the adenoma-carcinoma sequence is not known yet. Understanding the relationship between genetic and metabolic changes, as well as the role of these interactions in tumor initiation, is essential for designing efficient therapeutic approaches targeting the metabolism of tumors.

## 2 Materials and methods

### 2.1 Clinical material

All experiments were performed with human tissue samples. The present research protocol was approved by the Medical Research Ethics Committee (National Institute for Health Development, Tallinn, Estonia) by decisions number KK557 and KK558, and was following the Helsinki Declaration and Convention of the Council of Europe on Human Rights and Biomedicine. Research subjects were fully informed about the study and gave their consent.

Tumor and control tissue samples were obtained from the North Estonia Medical Centre. All patients (n=56 with ages ranging from 38 to 101) showed local or locally advanced disease (T2-4, N0-2), and only primary tumors were used. Normal tissue samples were taken from the same location at sites distant from the tumor and were checked for malignancies. Colorectal polyps were resected from patients (n=28, with ages ranging from 50 to 84) undergoing a colonoscopy at the West Tallinn Central Hospital. Only non-cancerous polyps were used. To maintain physiological conditions during geographical displacement, samples were placed immediately after removal into medium B (0.5 mM EGTA, 3 mM MgCl_2_, 60 mM K-lactobionate, 20 mM taurine, 3 mM KH_2_PO_4_, 110 mM sucrose, 0.5 mM dithiothreitol, 20 mM HEPES, 5 µM leupeptin, 2 mg/mL fatty acids free bovine serum albumin, pH 7.1). Additionally, a small amount of tissue was transported in RNALater Stabilization Solution (Qiagen).

### 2.2 Preparation of skinned tumor samples and permeabilization procedure

Upon arriving, samples were placed into pre-cooled (4°C) medium A consisting of 3 mM KH_2_PO_4_, 20 mM taurine, 5.7 mM ATP, 15 mM PCr, 9.5 mM MgCl_2_, 49 mM MES, 7.23 mM K_2_EGTA, and 2.77 mM K_2_CaEGTA, pH 7.1). Fat and blood vessels were removed from the tissue samples, which were then dissected into small samples (5-15 mg). These were permeabilized in medium A containing 50 µg/mL of saponin for 30 min at 4°C. The permeabilized samples were then washed three times for 5 min in pre-cooled medium B without leupeptin and kept at 4°C until use in oxygraphic analysis.

### 2.3 Oxygraphic measurements

The mitochondrial respiration of permeabilized tissue samples was measured in medium B at 25°C using a high-resolution respirometer Oxygraph-2k (Oroboros Instruments, Innsbruck, Austria). The medium was supplemented with 5 mM glutamate, 2 mM malate, and 10 mM succinate to fully activate respiratory chain complexes 1 and 2 (15). ADP was added in increasing concentrations to measure the dependence of respiration rate on exogenous ADP (**Supplementary S1**) and then calculate the apparent affinity of mitochondria to exogenous ADP (*K_m_* (ADP)). The obtained data were plotted as rates of O_2_ consumption (the basal respiration rate of respiration was subtracted) *versus* ADP concentration and *K_m_* (ADP) and *V_max_* values were calculated from these plots by nonlinear regression using Michaelis–Menten equation.

#### 2.3.1 Calibration of ADP stock solutions

To calibrate the concentration of ADP stock solution, the absorbance of NADH was determined using spectrophotometry. The reaction mixture contained a high K^+^ concentration medium (120 mM KCl, 20 mM MOPS, 1 mM EGTA, pH 7.2), 5 mM MgCl_2_, 1 mM phosphoenolpyruvate, 2,5 IU/mL lactate dehydrogenase, 3.75 IU/mL pyruvate kinase, and 0.15 mM NADH. The reaction was initiated by adding 1 µL of ADP stock and the concentration of ADP stock was defined as the decrease of NADH concentration. The extinction coefficient for NADH (6.22 x 10^3^ M^-1^ cm ^-1^) was used to convert its absorbance to molar concentration. *K_m_* (ADP) values were corrected accordingly.

### 2.4 RNA extraction

Tissue samples from patients were transported in RNALater solution (Qiagen) to protect cellular RNA until it was frozen in liquid nitrogen and stored at -80°C. The frozen tissue samples were homogenized by using the TRIzol reagent (Ambion). For RNA isolation, the RNeasy Mini Kit (Qiagen) was used by following the protocol by Untergasser (16). Genomic DNA was removed by using RNase-free DNase I solution (Qiagen). RNA was eluted in 30 µL of RNase-free water and the total concentration of RNA was measured by a BioSpec-Nano spectrophotometer (Shimadzu). Isolated RNA was stored at -80°C.

### 2.5 cDNA synthesis and real-time quantitative polymerase chain reaction

For cDNA synthesis and qRT-PCR, all reagents used were by Applied Biosynthesis. cDNA was synthesized from 2 µg of RNA by using a High-Capacity cDNA Reverse Transcription Kit with RNase inhibitor following the manufacturer’s instructions. Reverse transcription was performed with Eppendorf^®^ 5332 Mastercycler thermocycler.

qRT-PCR was performed with LightCycler 480 II (Roche) and by using the TaqMan Gene Expression Master Mix (Thermo Fisher Scientific). To detect gene expression levels, FAM-labeled TaqMan probes were used: actin-β (Hs01060665_g1), AK1 (Hs00176119_m1), AK2 (Hs01123132_g1), AK4 (Hs03405743_g1), AK6 (Hs00360444_g1), CK-BB (Hs00176483_m1), CK-MT1 (Hs00179727_m1), CK-MT2 (Hs00176502_m1), HK1 (Hs00175976_m1), HK2 (Hs00606086_m1), GLUT1 (Hs00892681_m1), LDHA (Hs03405707_g1), MCT1 (Hs00161826_m1), MCT2 (Hs04332706_m1), and MCT4 (Hs00358829_m1). MQ was used as a negative control.

### 2.6 DNA extraction

DNA was extracted from tissue samples using Invitrogen™ PureLink™ Genomic DNA Mini Kit following the instructions provided by the manufacturer. DNA concentrations and quality were measured using the NanoDrop 2000 spectrophotometer (Thermo Scientific, Waltham, MA, USA).

### 2.7 *KRAS* and *BRAF* mutation analysis

High-Resolution Melt (HRM) analysis was performed to detect the mutations in *KRAS* codon 12 and 13 of exon 2 and *BRAF* codon 600 of exon 15 (V600E). The reaction mix contained 1x HOT FirePol^®^ EvaGreen^®^ HRM Mix (Solis BioDyne, Estonia), 250 nM of sense and antisense primers (**Supplementary Table S2**), and 100x dilution of PCR amplification product. PCR amplification and HRM analysis were carried out with Rotor-Gene 6000 (QIAGEN) and consisted of an initial 15 min denaturation step at 95°C, followed by 45 cycles at 95°C for 10 s, 54°C for 10 s, and 72°C for 15 s, with a final extension at 72°C for 3 min. The obtained PCR products were heated at 95°C for 1 min and cooled down to 40°C to facilitate the formation of heteroduplex. HRM analysis was performed from 62°C to 92°C with a 0.1°C step. The results were analyzed using Rotor-Gene 6000 software and unknown samples were compared to control samples with known genotypes.

### 2.8 Data analysis

The authors confirm that the data supporting the findings of this study are available within the article and its **Supplementary Materials**. Data in text, figures, and tables are presented as mean ± standard error (SEM). Bar charts with individual data points were made by using SigmaPlot 11.0. The results from oxygraphic analysis and qRT-PCR were analyzed by Student’s *t*-test and p-values <0.05 were considered statistically significant. Apparent *K_m_* (ADP) values were measured by fitting experimental data to non-linear regression.

## 3 Results and discussion

### 3.1 Mitochondrial outer membrane permeability for ADP is different in healthy colon, polyps, and cancer tissue

To identify the changes in OXPHOS activity during the development of CRC, we applied high-resolution respirometry on permeabilized postoperative tissues (CRC, colon polyps, and healthy colon tissue). We determined the rate of maximal ADP-activated respiration (*V_max_*) and calculated the apparent Michaelis-Menten constant values for exogenously added ADP (*K_m_* (ADP)), to estimate the coupling of mitochondrial oxygen consumption to OXPHOS and the permeability of voltage-dependent anion channel (VDAC) for exogenous ADP, respectively.

Tissue or cell-specific tuning of OXPHOS activity through regulation of creatine, creatine-phosphate and adenine nucleotides movement *via* VDAC resulting in a certain *K_m_* (ADP) value could be a suitable indicator of the specific complexity of the intracellular organization, which is dealt with *ad hoc* isoforms of CK and AK for catalysis and intracellular energy transfer. In this regard, orders of magnitude different *K_m_* (ADP) values have been found between glycolytic and oxidative striated muscles (tissue-specific *K_m_* (ADP) values are higher in oxidative tissues), which have different metabolic features (17, 18). Thus, the determination of *V_max_* and *K_m_* (ADP) values for cellular oxygen consumption rates could provide relevant information about the activity of OXPHOS key components, the type of metabolism, and the complexity of the internal organization of the cells in the three different tissues included in the present study.

**Figure 1:**
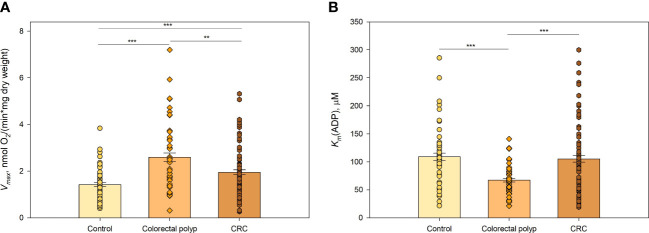
Kinetic parameters of mitochondrial respiration in normal tissue, colorectal polyps, and colorectal cancer tissue **(A)** Comparative analysis of maximal ADP-stimulated respiratory rate (*V_max_*); and **(B)** apparent Michaelis-Menten constant values for ADP (*K_m_*_(_ADP)) in control tissue (n=46), colorectal polyps (n=42), and colorectal cancer (CRC) tissue (n=57) ** p < 0,01, *** p < 0,001 (t-test).

There were significant differences in oxygen consumption *V_max_* and *K_m_* (ADP) values between polyps and tumors, suggesting that polyps and tumors have different bioenergetic profiles and demands for energy (**Figure 1**). The observation that *V_max_* for tumors was higher than for healthy colon tissue (**Figure 1A**; **Supplementary Table S3**) was in agreement with our previous studies (19–21). Interestingly, *V_max_* for colon polyps exceeded that in tumors and was two times higher than *V_max_* for healthy tissue. Determination of *K_m_* (ADP) values revealed that colon polyps have a significantly lower *K_m_* (ADP) compared to both cancerous and healthy tissue (**Figure 1B**). At the same time, healthy tissue and tumors showed similar *K_m_* (ADP) values, indicating a lower affinity for ADP than in polyps. The *V_max_/K_m_* (ADP) ratio was 0.039 min^-1^ mg^-1^ mL for colon polyps, whereas this ratio was similar and lower (0.019 and 0.013, respectively) for tumor and healthy tissue, indicating a more catalytically efficient system in polyps. Additionally, by calculating the % of mitochondrion with low oxidative capacity using the model developed by Saks and colleagues (22), polyps were characterized by a higher % of mitochondrion with low control over the movement of adenine nucleotides through MOM (**Supplementary Table S4**) compared to both healthy tissue and malignant tumors. This suggests that polyps have higher glycolytic capacity. These observations suggested a metabolic shift towards a more glycolytic type of metabolism while maintaining OXPHOS functionality in polyps, which was indicated respectively by the increased affinity for ADP on MIM and the high ADP-induced respiration level.

### 3.2 Polyps with *BRAF* mutation demonstrate a higher glycolytic activity together with some down-regulation of OXHOS

**Figure 2:**
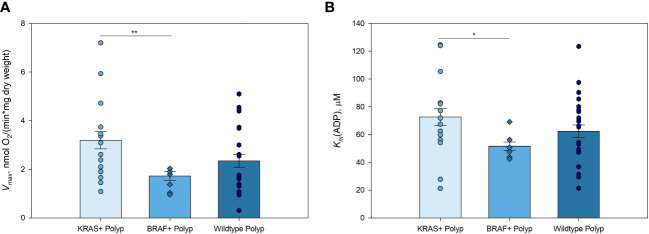
Kinetic parameters of mitochondrial respiration in *KRAS* and *BRAF* mutated, and wild-type polyps **(A)** Comparative analysis of maximal ADP-activated respiratory rate (*V_max_*); and **(B)** apparent Michaelis-Menten constant values for ADP (*K_m_* (ADP)) in *KRAS* mutated polyps (n=14), *BRAF* mutated polyps (n=6), and wild-type polyps (n=21) * p < 0,05, ** p < 0,01 (t-test).

The malignant transformation of cells, including colon epithelium, is accompanied by metabolic reprogramming of energy production and biosynthesis pathways that promote tumor growth and metastasis (23). Mutations in *KRAS* or *BRAF* genes appear to play a significant role in the transcriptional regulation of metabolic reprogramming in multiple cancers, including CRC (21, 24–27). The potential effect of *KRAS* and *BRAF* mutations on mitochondrial respiration was investigated in the colorectal polyp group (**Figure 2**).

Polyps with *KRAS* mutation showed higher *V_max_* values compared to those of polyps with *BRAF* mutation (**Figure 2A**; **Supplementary Table S5**). This pattern was similar to that obtained when comparing *BRAF* and *KRAS* mutations in CRC, with the difference that the *V_max_* of non-mutated CRC was higher than that in the tissue with *KRAS* and *BRAF* mutations (21). Mitochondria in *KRAS* mutated polyps showed lower affinity for exogenous ADP compared to that of *BRAF* mutated polyp group (**Figure 2B**). There were no significant differences in *V_max_* and *K_m_* (ADP), nor *V_max_/K_m_* (ADP) ratios, between *KRAS* or *BRAF* mutated and wild-type polyps. However, due to the significantly lower *V_max_* in both polyps and tumors with *BRAF* mutation, it may be assumed that cells with this mutation display a more active glycolysis with a parallel moderate down-regulation of OXPHOS. This metabolic profile of *KRAS* and *BRAF* mutated polyps suggested that energy metabolism during the transformation of polyps to colorectal cancer remain relatively unchanged. These results clearly need further assessment by using a larger study group.

To unveil respiratory rate kinetic parameters dependence on clinicopathological characteristics, possible relationships were analyzed of *V_max_* and *K_m_* (ADP) of polyps, tumors, and healthy colon tissue with age, gender, location, size, histological type, and molecular group (**Supplementary Table S5**). No relationship of *V_max_* and *K_m_* (ADP) values of healthy tissue, polyps, and CRC groups with clinicopathological factors was found. One exception was the location of polyps and tumors, which rendered different *V_max_* values. However, sample sizes among groups were unequal as CRC is more frequently observed in the distal than in the proximal area (28) and genetic architectures of proximal and distal CRC are partly distinct (29). Again, these results clearly require further assessment by using a larger study group.

### 3.3 Different gene expression levels show changes in energy metabolism during CRC tumorigenesis

Numerous genes and proteins essential for glucose uptake and glycolysis are upregulated in CRC and colon polyps (30–33). In the present study, RT-qPCR was performed to detect mRNA of genes coding for the glycolysis-controlling steps *GLUT1*, *HK1*, and *HK2* (8), as well as for the essential but not controlling steps *MCT1*, *MCT2*, *MCT4*, and *LDHA* and analyze their involvement in metabolic reprogramming in colon polyps. Absence of essential genes or proteins completely stops the functioning of the cellular process/function whereas fractional removal or inhibition of a controlling step brings about a corresponding decrease in the analyzed cellular process/function.

#### 3.3.1 Higher expression levels of genes coding for flux-controlling steps of glucose metabolism indicate an increased glycolytic activity in polyps

**Figure 3:**
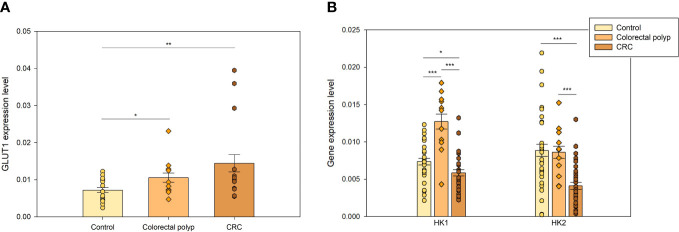
The expression levels of genes coding for flux-controlling steps in glycolysis **(A)** GLUT 1 expression levels in healthy colon (n=16), colorectal polyps (n=8), and colorectal tumors (n=9) **(B)** Expression levels of *hexokinase 1* (*HK1*) and *hexokinase 2* (*HK2*) in healthy tissue (n=27), colorectal polyps (n=9), and colorectal cancer (CRC) tissue (n=27). * p < 0,05, ** p < 0,01, *** p < 0,001.

The first step in glucose metabolism is the entrance of glucose into the cell, which relies on glucose transport proteins (GLUTs). GLUTs belong to a homologous family of fourteen uniporter transporter proteins. Among these, GLUTs 1-4 have been extensively studied and shown to be upregulated in cancers (34). There is an increasing number of studies identifying GLUT1 (glucose low affinity isoform) and GLUT3 (glucose high affinity isoform) as preeminent actors in accelerated glucose metabolism. High expression of *GLUT1* is associated with poor survival in most cancer types, including colorectal cancer (35). The lower *GLUT1* expression levels in control colon tissue (**Figure 3A**) compared to diseased states were consistent with the notion that glucose provides a smaller fraction of the energy requirements for the healthy colonic epithelium. The expression of GLUT1 in colorectal polyps was significantly higher than in normal tissue, suggesting an increased demand for glucose. The CRC group also showed an increased level of *GLUT1* expression compared to the healthy colon tissue. The polyp group showed a tendency towards a lower expression of *GLUT1* than the CRC group but there was no significant difference (p=0.136). An increase in glucose uptake may indicate significant changes in energy metabolism as well as in anabolic precursors demand such as glucose-6-phosphate for pentose phosphate pathway, dihydroacetonephosphate for triacylglyceride and phospholipid syntheses, and 3-phosphoglycerate for serine, cysteine and glycine syntheses occurring in the tissue at early events of carcinogenesis.

Hexokinase (HK) is a flux-controlling step of glycolysis catalyzing ATP-dependent phosphorylation of glucose into glucose-6-phosphate (8). Four major HK isoforms, encoded by separated genes, are expressed in human tissues – HK1-4 (36). HKs help to sustain cellular glucose levels by regulating the entry and utilization of glucose and influencing the magnitude and the direction of glucose flux within cells (37). HK1 is the predominant HK isoform in most tissues, is a glucose high affinity isoform and is more abundant than HK2. HK2 is a glucose low affinity isoform and the main isoenzyme in insulin-sensitive organs such as heart, skeletal muscle, and adipose tissue, and in a wide range of tumors.

HK1 and HK2 can also dock to mitochondria through an N-terminal motif absent in the other isoforms. When bound to mitochondria, HK1 and HK2 exert cytoprotective effects in healthy and neoplastic cells and increase their efficiency in glucose usage (38). Pedersen proposed that HK2 promotes the Warburg effect by binding to VDAC (11). This interaction leads VDAC to redirect mitochondrial ATP to HK2 to be used in glycolysis. Thus, HK has been proposed to regulate the MOM permeability in glycolytic cancer cells (14, 39). The high expression level and activity of *HK2* together with that of GLUTs in glycolytic cancers are indirectly revealed by ^18^FDG-PET imaging (38).

Expression of *HK1* and *HK2* was analyzed in healthy colon tissue, colorectal polyp, and CRC groups. *HK1* expression level in polyps was twice as high as in healthy tissue and CRC group, while their *HK2* expression was similar to that of healthy tissue group (**Figure 3B**). Distinct differences in the regulation of mitochondrial respiration in polyps (**Figure 1**), specifically lower *K_m_* (ADP) values, suggested that polyps have a more glycolytic type of regulation of energy metabolism than CRC and healthy tissue. *HK2* overexpression has been previously shown in CRC cells, in comparison to normal cells (33, 40). However, our data did not reveal *HK2* overexpression in the CRC group. In fact, it was significantly lower than that of healthy tissue (p<0.001). Then, the low *HK2* expression levels in the CRC group suggested that energy metabolism in CRC cells was not entirely glycolytic and that OXPHOS system was an important energy provider.

In turn, higher expression levels of *HK1* and *HK2* in polyps compared to the CRC group suggested that glycolytic metabolism played a more essential role in polyps than in CRC. Considering the moderate *GLUT1* expression levels and the ensuing moderate glucose uptake in polyps (**Figure 3A**), then *HK* overexpression seemed counterproductive. We speculate that there was no need to remarkably increase the glucose uptake mediated by GLUT1 in polyps, because highly expressed *HK*s, and perhaps GLUT3, were able to drive an enhanced glycolytic flux.

#### 3.3.2 Expression levels of genes coding for essential but non-controlling steps support the conclusion that polyps increase glycolytic activity

**Figure 4:**
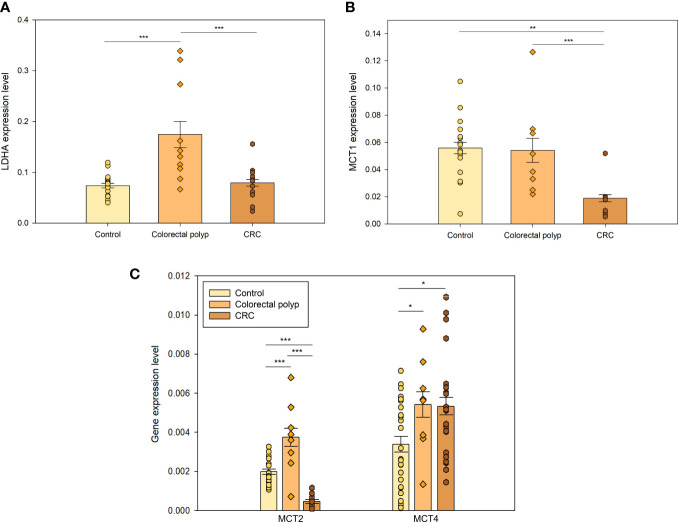
The expression levels of essential but non-controlling steps in glycolysis **(A)** The expression levels of *lactate dehydrogenase A* (*LDHA*) in healthy tissue (n=16), colorectal polyps (n=8), and colorectal cancer (CRC) tissue (n=9). **(B)** The xpression levels of monocarboxylate transporters *MCT1* and **(C)** *MCT2* in healthy tissue (n=16), colorectal polyps (n=8) and colorectal cancer (CRC) tissue (n=9); and expression levels of *MCT4* in healthy tissue (n=24), colorectal polyps (n=8) and CRC tissue (n=22) * p < 0,05, ** p < 0,01, *** p < 0,001 (t-test).

Lactate dehydrogenase A (LDHA) is an essential enzyme in the glycolytic pathway that catalyzes the conversion of pyruvate to lactic acid using NADH and recycling NAD+. Elevated levels of this protein have been found in several cancer types (41, 42), supporting cancer cell proliferation and survival. The low *K_m_* (ADP) value (**Figure 1B**) and high expression levels of *HK*s (**Figure 3B**) suggested that cells in colon polyps developed an increased dependence on the glycolytic pathway. This was further supported by the elevated levels of *LDHA* expression in polyps compared to the healthy colon tissue (**Figure 4A**), suggesting a rise in lactate production. Although LDH is not a flux-controlling step of glycolysis since it is one of the fastest pathway steps, it exerts full control on the pyruvate and lactate levels and hence on the cytosolic redox balance (the Pyr/Lac ratio is tightly linked to the NADH/NAD+ ratio by the overexpressed high LDH activity).

Moreover, increased production of lactate and its further release together with H^+^ promotes malignant progression by lowering extracellular pH, which helps cancer cells to overcome host immune response (43). In addition, aerobic glycolysis does not only supply ATP, but also yields metabolic precursors for nucleotides, amino acids, and lipids biosynthesis for cell proliferation (44). Therefore, the high expression of *LDHA* in polyps helps promoting the disease progression to malignancy. There was no significant difference between the expression levels of *LDHA* between the CRC and the healthy tissue group, suggesting that CRC cells do not need to increase lactate production and the ensuing external acidification further because they mainly increase OXPHOS for energy supply and expression of glycolytic controlling steps for anabolic precursors and pyruvate provision.

Healthy colonocytes derive 60-70% of their energy supply from short-chain carboxylic acids, particularly butyrate. Butyrate is transported across the luminal membrane of the colonic epithelium *via* a monocarboxylate transporter (MCT1) (45). MCT1 is a member of the monocarboxylate transporter family, of which 14 isoforms have been identified. In the present study, the expression of *MCT1*, *MCT2*, and *MCT4* was analyzed. Healthy colon tissue showed higher expression level of *MCT1* compared to the tumor (**Figure 4B**), which is aligned with the fact that butyrate is the main source of energy for colonic epithelial cells (46). Decreased *MCT1* expression in cancer tissue could indicate that cancer cells use less butyrate, displaying metabolic plasticity and making them less dependent on this nutrient.

MCT1 has a high affinity for extracellular lactate and has been shown to transport lactate to sustain energy production in malignant cells (47). Therefore, its low expression level in tumors suggested that CRC cells did not depend on lactate as a metabolic fuel. *MCT1* was expressed in colon polyps similarly to healthy tissue but the expression levels of isoforms *MCT2* and *MCT4* were increased (**Figure 4C**). In highly glycolytic cancer cells, MCT2 has been shown to localize mainly in the cytosol (48). Decreased expression of *MCT2* in the CRC group compared to the healthy colon tissue group again supported the idea that CRC did not acquire a typical Warburg effect. MCT4 has a low affinity for extracellular lactate and high affinity for intracellular lactate, as well as very high activity for lactate transport and a very low affinity for pyruvate (48), meaning that pyruvate is rather converted to lactate than transported out of the cell whereas internal lactate can be actively expelled.

Similar expression level of *MCT1* (**Figure 4B**) in control and polyp groups suggested that colon polyps kept using short-chain carboxylic acids, and perhaps other substrates (*e.g*. glutamine). However, polyps exhibited higher *MCT4* expression (**Figure 4C**) than control indicating that they simultaneously increased glycolytic activity. *MCT4* is upregulated by hypoxia and hypoxia-inducible factor 1alpha (HIF-1*alpha*) (49). It has been shown that *HIF-1alpha* levels are increased in colon polyps and CRC (50). There is a steep oxygen gradient from the anaerobic lumen of the intestine across the epithelium into the highly vascularized sub-epithelium. Epithelial cells lining the mucosa are exposed to a relatively low O_2_ tension environment that has been described as “physiological hypoxia (51, 52).” From this perspective, it is perhaps not surprising to see overexpression of *MCT4* in colon polyps as energy demand increases while there is still a low level of oxygenation.

#### 3.3.3 Intracellular phosphotransfer pathways are upregulated in colon polyps

Adenylate kinase (AK) and creatine kinase (CK) play an important role in adjusting mitochondrial ATP synthesis to cellular ATP consumption by forming phosphotransfer circuits, which connect sites of ATP production (glycolysis and OXPHOS) with subcellular sites of ATP utilization (ATPases) to support robust metabolic homeostasis (53–55).

AKs catalyze the reversible interconversion of adenine nucleotides (AMP, ADP, ATP), and they represent the main mediator of intracellular nucleotide exchange and AMP metabolic signaling (56). Suppression of AK phosphotransference and AMP generation in cancer cells, and consequently signaling through AMPK, might be a triggering factor in the initiation of malignant transformation, unleashing uncontrolled cell cycle turnover and proliferation (57). Nine different adenylate kinase isoenzymes (AK1-9) have been identified and characterized so far in human tissues, displaying different organ and subcellular distributions.

**Figure 5:**
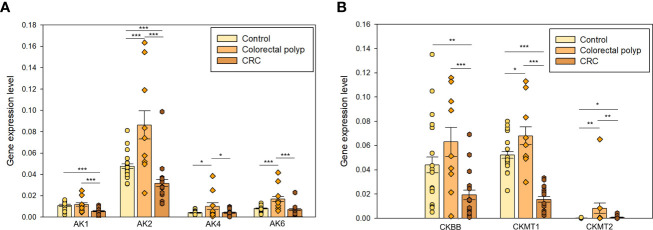
The expression levels of **(A)** adenylate kinases *AK1*, *AK2*, *AK4*, and *AK6;* and creatine kinases **(B)** *CKBB, CKMT1, and CKMT2* in the healthy colon (n=19), colorectal polyps (n=9), and colorectal cancer tissue (n=15) * p < 0,05, ** p < 0,01, *** p < 0,001 (t-test).

In the present study, the gene expression level of *AK1*, *AK2*, *AK4*, and *AK6* was analyzed. AK1 is expressed in the cytosol at high levels in brain, heart, skeletal muscles, and erythrocytes (58). Previous studies have shown that AK activity in CRC tumor tissue is higher than in normal mucosa (59). AK1 has been proposed to be a negative regulator of colorectal cancer development. Its expression level in the polyp group was like that found in healthy tissue and significantly higher compared to the CRC group (**Figure 5A**).

AK2 isoform is localized in the mitochondrial intermembrane space and regulates the ATP/ADP transference rate between the cytosol and mitochondrial matrix (56). Changes in the regulation of AK2 have been observed in several human cancers. *AK2* overexpression has been observed in lung adenocarcinoma, triple-negative breast cancer cells, and neuroblastoma cell lines and it could be related to the aggressive nature of these cancer types (60–62). The expression of AK2 was upregulated in the polyp group compared to CRC and healthy tissue groups (**Figure 5A**). The CRC group showed a significantly lower *AK2* expression than the control group suggesting that fundamental rearrangements in the energy-related communication networks between cytosol and mitochondria take place during progression to cancer. Although the potential role of AK2 in tumorigenesis has been reported for a long time already, its underlying mechanism is still unclear.

*AK4* is expressed in the mitochondrial matrix and may indirectly modulate the mitochondrial membrane permeability *via* its interactions with the ADP/ATP translocase (ANT) (58). Previous studies have demonstrated the involvement of AK4 in the progression of different cancer types, as well as in the resistance to radiation therapy and multiple chemotherapeutic agents (63–65). Indeed, the expression level of *AK4* was significantly higher in the polyp group compared to the healthy tissue group and CRC. AK4 has been demonstrated to promote a glycolytic shift (66), which is aligned with our observation of the glycolytic phenotype in the polyp group.

AK6, renamed as human coilin interacting nuclear ATPase protein (HCINAP), is localized in nucleus and cytosol, and is ubiquitously expressed in different tissues and cell types (67, 68). *AK6* expression was higher in polyps than in control tissue and CRC groups (**Figure 5A**). AK6 is a glycolysis regulator *via* phosphorylation of LDHA and a modulator of invasion and metastatic activity of cancer stem cells (69). However, *AK6* became upregulated already at the polyp stage and it may support the glycolytic activity in benign tumors. Higher *LDHA* expression level in polyps than CRC (**Figure 4A**) correlates with a similar pattern in *AK6* expression. It can be hypothesized that AK6 is required to support cell division and that in polyps with active anaerobic glycolysis AK6 could be preferentially located in the cytosol.

Moreover, AKs may regulate intracellular AMP levels and thus directly affect AMPK metabolic signaling. The elevated levels of AK expression in polyps could support AMPK activation and OXPHOS in conditions of intense competition for cytosolic ADP (glycolysis has a high affinity for ADP). In this regard, a model has been developed where the bioenergetics signatures combine the metabolic networks of AMPK and HIF-1 alpha activity (70). The activity of these two regulators defines the metabolic states as follows: a glycolytic state is established by high HIF-1 and low AMPK, an oxidative state is characterized by low HIF-1 and high AMPK, and in a hybrid state both regulators are active (70). As both proteins are important determinants of cell metabolism and fate, understanding the interplay between different AKs in their various locations and their regulation might uncover new targets for cancer treatments or biomarkers for cancer occurrence and prognosis.

Creatine kinase (CK) has a crucial role in cell bioenergetics to efficiently regenerate ATP from phosphocreatine and is over-expressed in cells with high energy requirements such as skeletal, cardiac and smooth muscle, kidney, and brain (71). There are two genes for cytosolic CK subunit isoforms forming three types of dimers (CKMM, CKBB, and CKMB) and two mitochondrial creatine kinase (mtCK) isoenzymes (the *ubiquitous* form – gene *CKMT1* and the *sarcomeric* form – gene *CKMT2* (72). The interplay between cytosolic and mitochondrial CK isoenzymes depends on a large intracellular pool of creatine/phosphocreatine and prevents a rapid fall in global ATP concentrations (72). This ATP buffering system is known as the phosphocreatine (PCr)-creatine kinase (CK) shuttle, or PCr-CK circuit (53).

Mitochondrial CKs catalyze the interconversion of ATP into PCr at the main ATP-producing sites to store the energy in the form of PCr and facilitate its intracellular diffusion across the different subcellular organelles, whereas cytosolic CKs regenerate *in situ* ATP from the PCr pool at ATP-consuming sites (73, 74). CKs are expressed in colon epithelial cells and are coordinately regulated by HIFs. Such regulation is critical for their barrier function (75). Attenuated expression of CK enzymes in inflammatory bowel disease tissue (75), downregulation of CK-BB functional activity and low expression of *MTCK1* in colon cancer (which is a different feature from other cancer types) (59, 76) suggest that intestinal creatine metabolism and PCr/CK circuit may be compromised in colon polyps as well.

Here, the expression level of *CKBB*, *CKMT1*, and *CKMT2* was assessed. Downregulation of *CKBB* in CRC was observed (**Figure 5B**). CK isoforms may be up- and down-regulated in tumors depending on the nature of the carcinogenesis (77). Mitochondrial CK transcribed from *CKMT1*, also known as U-MtCK is localized in the inner membrane of mitochondria. CKMT1 may participate in the development of human cancers because of its involvement in several cellular processes such as cell proliferation, migration, and apoptosis (78, 79). Expression of *CKMT1* was significantly lower in the CRC group compared to healthy tissue and polyps (**Figure 5B**). In this regard, it has been suggested that *MtCK* expression is regulated by the metabolic energy cell status and their expression may represent a mechanism to compensate for a low energy state (72). Thus, high expression of *CKBB* and *CKMT1* in control and polyp, and overexpression of *CKMT2* in polyps is consistent with the observation that polyps are highly glycolytic compared to healthy and cancerous tissue. Whether the changes in intracellular energy transfer are the cause or consequence of CRC and hence how AK and CK energy shuttles may be affected to prevent polyps from becoming malignant remains to be investigated.

## 4 Conclusions

**Figure 6:**
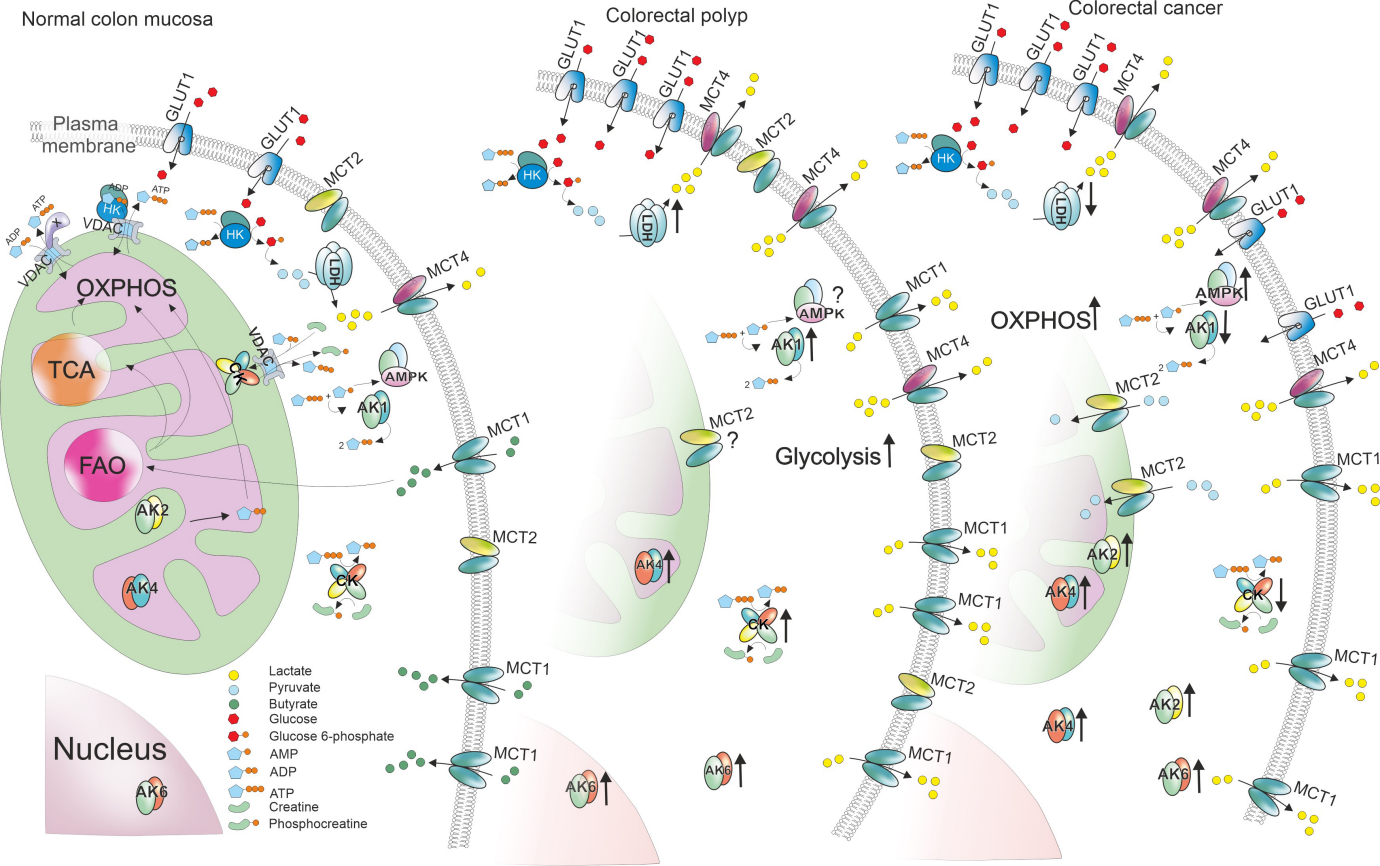
Changes of gene expression levels in colorectal polyps and tumors compared to normal colon mucosa. ATP production in normal colon mucosa is presented in the left. Glucose enters the cell through glucose transporters (GLUT1) and is then phosphorylated by hexokinase (HK). The glycolytic product pyruvate enters mitochondria through voltage dependent anion channel (VDAC) in mitochondrial outer membrane and is used to produce ATP through TCA cyle and oxidative phosphorylation (OXPHOS). Some of the pyruvate is converted into lactate by lactate dehydrogenase A (LDHA) and lactate is transported out of the cell using monocarboxylate transporter (MCT) 4. Additionally, colon cells uptake short fatty acids like butyrate using MCT1 and use them in fatty acid oxidation (FAO). To transport ATP and ADP between energy production and energy consumption sites, cells use creatine kinases (CKs) and adenylate kinases (AKs). In colorectal polyps, the energy metabolism is reprogrammed by increasing the glycolytic activity. This phenomenon could be facilitated by higher *GLUT1*, *HK* and *LDHA* expression boosting lactate production. The excessive amounts of lactate are transported out of the cell using MCT1 and MCT4 that were upregulated in polyps. Polyps also displayed higher levels of both CKs and AKs. However, colorectal cancer cells (right) seemed to depend more on OXPHOS suggested by the decreased expression levels of *HK*s and *LDHA*. Poor *MCT* levels indicate that the need for lactate transport is lower. Opposite to polyps, energy transport pathways in cancer cells showed signs of downregulation.

Although our knowledge on cancer metabolism has increased, the whole process of metabolic reprogramming during tumorigenesis is still rather unexplored. Here we showed that changes in energy production already occur in benign colorectal tumors and the alterations continue throughout the development of colorectal cancer. Colon polyps seem to increase glycolytic activity by overexpressing glucose transporter 1 and hexokinases. The low *K_m_* (ADP) value determined in polyps by high-resolution respirometry as well as their LDHA overexpression added support to the proposal of a glycolytic phenotype for polyps. The higher glycolytic activity may drive cell proliferation in the diseased state (80). On the other hand, while cancer cells seem to upregulate the glycolytic pathway, they still depend highly on mitochondrial respiration. Besides glycolysis, colon polyps upregulate the activity of energy transfer pathways like adenylate kinase and creatine kinase systems. The observations of metabolic reprogramming described in the results are presented in **Figure 6**. The significant changes in gene expression levels could be used as biomarkers to detect benign tumors in early stages.

## Data availability statement

The raw data supporting the conclusions of this article will be made available by the authors, without undue reservation.

## Ethics statement

The studies involving human participants were reviewed and approved by Research Ethics Committee of the National Institute for Health Development. The patients/participants provided their written informed consent to participate in this study.

## Author contributions

ER-K, LR and MP wrote the main manuscript text. ER-K and LR performed the experiments and analyzed the data. LR and IS prepared the figures. JB, KS and VV provided the samples. IS prepared the graphical abstract. IS, RM-S and TK reviewed and edited the text. All authors contributed to the article and approved the submitted version.
