# Supplementary material for: Colorectal polyps increase the glycolytic activity
Source: Front Oncol. 2023 Jun 5;13:1171887. doi: 10.3389/fonc.2023.1171887 (PMC10277630; doi:10.3389/fonc.2023.1171887)
Supplement: Supplementary file 1 [file Table_1.docx]

Supplementary Material

# Supplementary data S1

**Examples of the original traces of O_2_ consumption by permeabilized tissue upon the addition of increasing concentrations of ADP in polyp (A), tumor (B), and healthy tissue (C).** The red line shows the O_2_ flux per sample mass, the blue line shows the O_2_ consumption. First, respiratory complexes I and II were activated by the addition of Glutamate (Glut), Malate (Mal), and Succinate (Succ) [1]. ADP was added in increasing concentration (25 μM – 1 mM) until maximum respiration was achieved. **(D)** The measured rates of respiration for samples A, B, and C plotted against respective ADP concentrations. The apparent *K_m_*(ADP) and *V_max_* were calculated by nonlinear regression using the Michaelis-Menten equation. The visual representation of how *K_m_*(ADP) and *V_max_* values are determined is only shown for the polyp. The upper dashed line shows *V_max_* for the polyp sample and *K_m_*(ADP) is the ADP concentration at which the velocity of the reaction is half of *V_max_*.


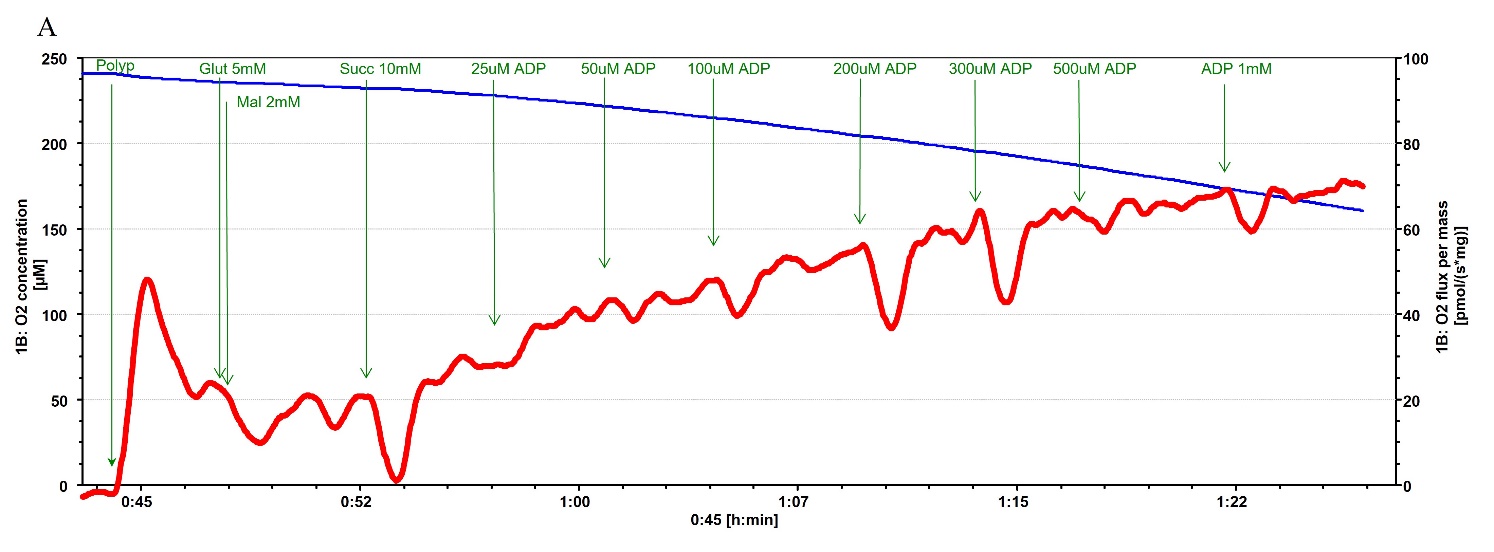


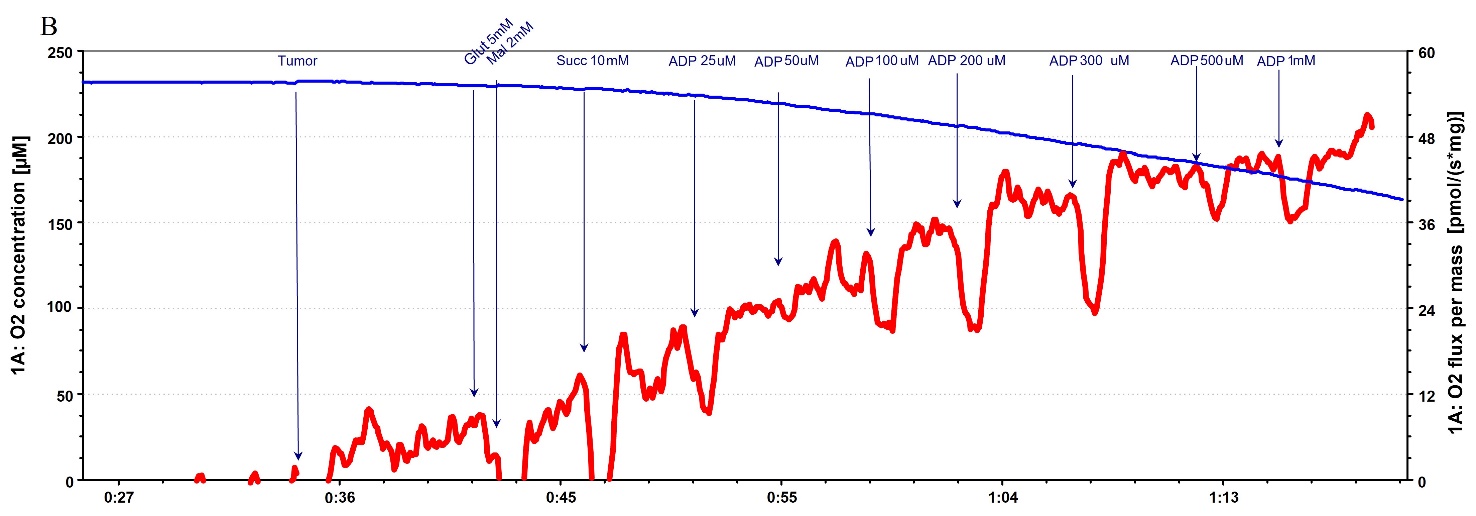


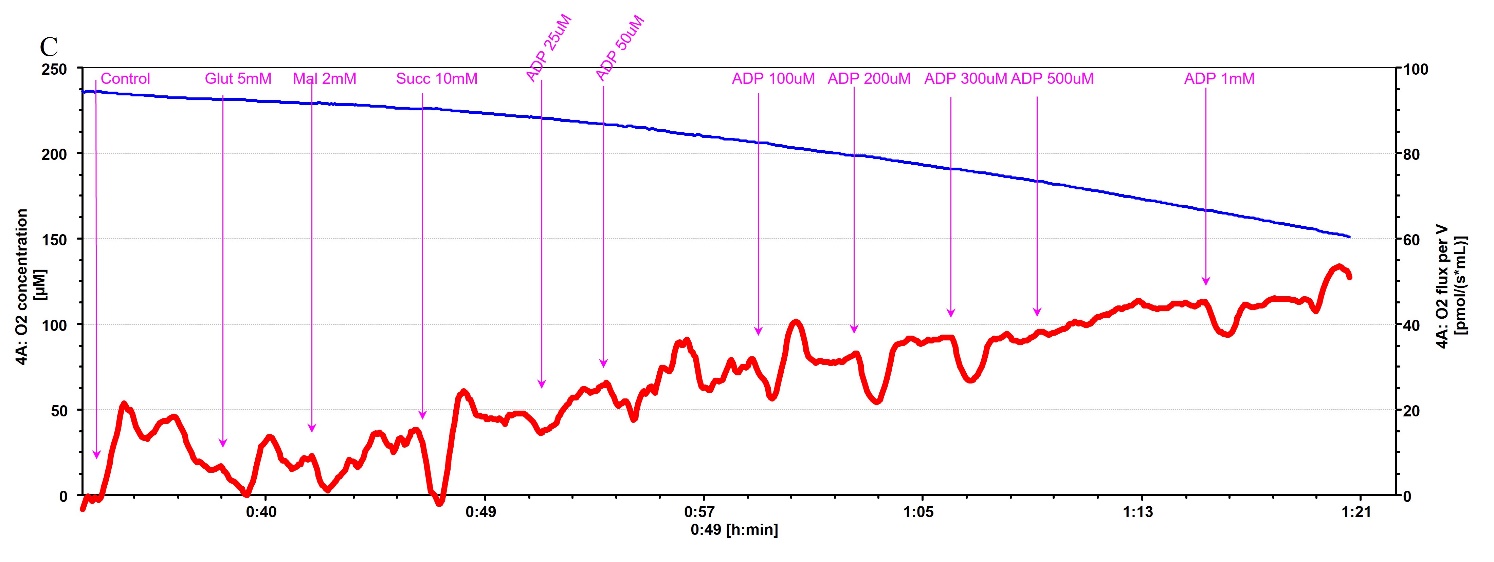


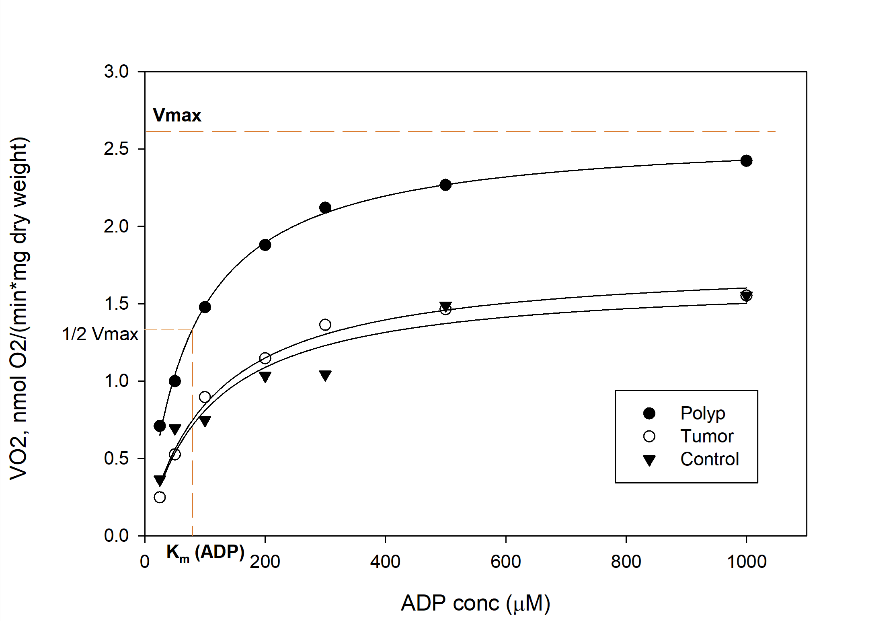


D

1. Pesta, D. and E. Gnaiger, *High-Resolution Respirometry: OXPHOS Protocols for Human Cells and Permeabilized Fibers from Small Biopsies of Human Muscle*, in *Mitochondrial Bioenergetics: Methods and Protocols*, C.M. Palmeira and A.J. Moreno, Editors. 2012, Humana Press: Totowa, NJ. p. 25-58.
