# Supplementary material for: Colorectal polyps increase the glycolytic activity
Source: Front Oncol. 2023 Jun 5;13:1171887. doi: 10.3389/fonc.2023.1171887 (PMC10277630; doi:10.3389/fonc.2023.1171887)
Supplement: Supplementary file 2 [file Table_2.docx]

Supplementary Material

# Supplementary Table S2

**Primers used for HRM analysis**

| Primer | Sequence |
| --- | --- |
| *KRAS*-antisense | 5′- AAATGACTGAATATAAACTTGTGGTAGT-3′ |
| *KRAS*-sense | 5′- TGAATTAGCTGTATCGTCAAGGCACT-3′ |
| *BRAF-*antisense wild-type | 5′-cgccgcgcgccAAAATAGGTGATTTTGGTCT-3′ |
| *BRAF*-antisense mutation | 5′-TAAAAATAGGTGATTTTGGTCTAGCTACA-3′ |
| *BRAF*-sense | 5′- CCACAAAATGGATCCAGACAACTG 3′ |
