# Supplementary material for: Colorectal polyps increase the glycolytic activity
Source: Front Oncol. 2023 Jun 5;13:1171887. doi: 10.3389/fonc.2023.1171887 (PMC10277630; doi:10.3389/fonc.2023.1171887)
Supplement: Supplementary file 3 [file Table_3.docx]

Supplementary Material

# Supplementary Table S3

**Comparison of the maximal ADP-stimulated respiration rate (*V_max_*) and apparent Michaelis-Menten constant for exogenously added ADP (*K_m_*(ADP)) in human tumors, polyps, and control colon tissue.**

|  | *V_max_*,  nmol O_2_/(min×mg dry weight) | *K_m_*(ADP),  µM |
| --- | --- | --- |
| Control (n=46) | 1.43 ± 0.08 | 109.06 ± 6.25 |
| Tumor (n=68) | 1.95 ± 0.10 | 105.26 ± 5.94 |
| p-value | <0.001 | 0.669 |
|  |  |  |
| Control (n=46) | 1.43 ± 0.08 | 109.06 ± 6.25 |
| Polyp (n=45) | 2.60 ± 0.18 | 67.23 ± 3.30 |
| p-value | <0.001 | <0.001 |
|  |  |  |
| Tumor (n=68) | 1.95 ± 0.10 | 105.26 ± 5.94 |
| Polyp (n=45) | 2.60 ± 0.18 | 67.23 ± 3.30 |
| p-value | 0.001 | <0.001 |
