# Supplementary material for: Colorectal polyps increase the glycolytic activity
Source: Front Oncol. 2023 Jun 5;13:1171887. doi: 10.3389/fonc.2023.1171887 (PMC10277630; doi:10.3389/fonc.2023.1171887)
Supplement: Supplementary file 4 [file Table_4.docx]

Supplementary Material

# Supplementary Table S4

**The modeled % of the low oxidative capacity mitochondrion in control, colon polyp, and CRC tissue groups.** The mathematical model to calculate the percentage of the low oxidative capacity mitochondrion was developed by Saks, V., et al [1] in muscle tissue. They calculated average *K_m_* values and plotted these against the fraction of low *K_m_* population of mitochondria in the sample showing that *K_m_* can be used to calculate the ratio of different mitochondrial populations. Based on the calculation from this study:

$$Oxidative capacity of mitochondrion \left( \% \right)=142.2879\times exp(-K_{m}\times0.0139)$$

| **Sample group** | **% of the low oxidative capacity mitochondrion** |
| --- | --- |
| Control (n=46) | 31.2 |
| Colon polyps (n=45) | 55.9 |
| CRC (n=68) | 32.9 |

1. Saks, V., et al., *Permeabilized cell and skinned fiber techniques in studies of mitochondrial function in vivo.* Molecular and Cellular Biochemistry, 1998. **184**: p. 81-100.
