# Supplementary material for: Colorectal polyps increase the glycolytic activity
Source: Front Oncol. 2023 Jun 5;13:1171887. doi: 10.3389/fonc.2023.1171887 (PMC10277630; doi:10.3389/fonc.2023.1171887)
Supplement: Supplementary file 5 [file Table_5.docx]

Supplementary Material

# Supplementary Table S5

**Clinicopathological factors of included patients with tumors and polyps.**

| **Variable** | **Patients** | **Control**  (n = 46) | | **Patients** | **Tumor**  (n = 56) | | **Patients** | **Polyp**  (n = 28) | |
| --- | --- | --- | --- | --- | --- | --- | --- | --- | --- |
|  |  | *V_max_* | *K_m_(ADP)* |  | *V_max_* | *K_m_(ADP)* |  | *V_max_* | *K_m_(ADP)* |
| Age |  |  |  |  |  |  |  |  |  |
| <65 y | 12 | 1.55 ± 0.18 | 115.4 ± 9.0 | 14 | 2.14 ± 0.17 | 122.0 ± 16.2 | 6 | 3.41 ± 0.36 | 56.6 ± 7.1 |
| >65 y | 34 | 1.39 ± 0.09 | 106.8 ± 7.9 | 41 | 1.88 ± 0.14 | 102.6 ± 6.4 | 14 | 2.95 ± 0.33 | 68.8 ± 6.5 |
| P-value |  | 0.401 | 0.554 |  | 0.299 | 0.182 |  | 0.427 | 0.286 |
|  |  |  |  |  |  |  |  |  |  |
| Gender |  |  |  |  |  |  |  |  |  |
| Female | 24 | 1.41 ± 0.13 | 110.6 ± 9.7 | 28 | 1.83 ± 0.15 | 113.8 ± 8.2 | 8 | 2.04 ± 0.25 | 48.6 ± 5.0 |
| Male | 22 | 1.45 ± 0.11 | 107.4 ± 7.9 | 28 | 2.06 ± 0.14 | 97.7 ± 8.2 | 17 | 2.64 ± 0.27 | 68.2 ± 4.2 |
| P-value |  | 0.824 | 0.801 |  | 0.266 | 0.170 |  | 0.020 | 0.227 |
|  |  |  |  |  |  |  |  |  |  |
| Location |  |  |  |  |  |  |  |  |  |
| Proximal | 27 | 1.42 ± 0.11 | 106.1 ± 8.1 | 31 | 1.66 ± 0.14 | 99.0 ± 6.7 | 6 | 3.78 ± 0.74 | 66.7 ± 12.3 |
| Distal | 19 | 1.44 ± 0.13 | 113.3 ± 9.9 | 25 | 2.26 ± 0.14 | 111.9 ± 10.6 | 19 | 2.19 ± 0.18 | 62.7 ± 3.6 |
| P-value |  | 0.874 | 0.572 |  | 0.004 | 0.302 |  | 0.004 | 0.673 |
|  |  |  |  |  |  |  |  |  |  |
| T stage |  |  |  |  |  |  |  |  |  |
| I-II stage | NA | NA | NA | 28 | 1.80 ± 0.11 | 92.4 ± 8.0 | NA | NA | NA |
| III-IV stage | NA | NA | NA | 17 | 1.73 ± 0.25 | 135.1 ± 12.9 | NA | NA | NA |
| P-value |  |  |  |  | 0.800 | 0.005 |  |  |  |
|  |  |  |  |  |  |  |  |  |  |
| Histology |  |  |  |  |  |  |  |  |  |
| Tubular type | NA | NA | NA | NA | NA | NA | 5 | 2.36 ± 0.25 | 59.9 ± 3.2 |
| Tubulovillous and villous type | NA | NA | NA | NA | NA | NA | 6 | 3.79 ± 0.49 | 64.5 ± 11.6 |
| P-value |  |  |  |  |  |  |  | 0.009 | 0.593 |
|  |  |  |  |  |  |  |  |  |  |
| KRAS status |  |  |  |  |  |  |  |  |  |
| Wild-Type | NA | NA | NA | 20 | 1.97 ± 0.16 | 96.3 ± 9.9 | 15 | 2.35 ± 0.27 | 62.5 ± 4.5 |
| KRAS mutation | NA | NA | NA | 15 | 1.89 ± 0.13 | 95.3 ± 8.7 | 7 | 3.19 ± 0.35 | 72.6 ± 6.1 |
| P-value |  |  |  |  | 0.693 | 0.941 |  | 0.062 | 0.182 |
|  |  |  |  |  |  |  |  |  |  |
| BRAF status |  |  |  |  |  |  |  |  |  |
| Wild-Type | NA | NA | NA | 20 | 1.97 ± 0.16 | 96.3 ± 9.9 | 15 | 2.35 ± 0.27 | 62.5 ± 4.5 |
| BRAF mutation | NA | NA | NA | 7 | 1.38 ± 0.30 | 82.8 ± 9.8 | 3 | 1.52 ± 0.16 | 51.7 ± 3.0 |
| P-value |  |  |  |  | 0.069 | 0.440 |  | 0.120 | 0.227 |

NA* - no information
